# Polarized Growth in the Absence of F-Actin in *Saccharomyces cerevisiae* Exiting Quiescence

**DOI:** 10.1371/journal.pone.0002556

**Published:** 2008-07-02

**Authors:** Annelise Sahin, Bertrand Daignan-Fornier, Isabelle Sagot

**Affiliations:** 1 Université de Bordeaux - Institut de Biochimie et Génétique Cellulaires, Bordeaux, France; 2 CNRS – UMR5095, Bordeaux, France; Wellcome Trust Sanger Institute, United Kingdom

## Abstract

**Background:**

Polarity establishment and maintenance are crucial for morphogenesis and development. In budding yeast, these two intricate processes involve the superposition of regulatory loops between polarity landmarks, RHO GTPases, actin-mediated vesicles transport and endocytosis. Deciphering the chronology and the significance of each molecular step of polarized growth is therefore very challenging.

**Principal Findings:**

We have taken advantage of the fact that yeast quiescent cells display actin bodies, a non polarized actin structure, to evaluate the role of F-actin in bud emergence. Here we show that upon exit from quiescence, actin cables are not required for the first steps of polarized growth. We further show that polarized growth can occur in the absence of actin patch-mediated endocytosis. We finally establish, using latrunculin-A, that the first steps of polarized growth do not require any F-actin containing structures. Yet, these structures are required for the formation of a *bona fide* daughter cell and cell cycle completion. We propose that upon exit from quiescence in the absence of F-actin, secretory vesicles randomly reach the plasma membrane but preferentially dock and fuse where polarity cues are localized, this being sufficient to trigger polarized growth.

## Introduction

The ability to grow asymmetrically is essential for a large variety of cellular processes such as cell division or migration, and is therefore crucial for morphogenesis and development. For years, *Saccharomyces cerevisiae*, which undergoes polarized growth during various phases of its life cycle, has been a model of choice for studying the molecular mechanisms underlying polarity establishment. Budding yeast is an attractive model since it has a predictable polarization pattern. Further, in *Saccharomyces cerevisiae*, by contrast with other organisms, the polarized delivery of secretory vesicles is mediated by the actin cytoskeleton and microtubules do not appear to be involved in this process [1], [2].

In budding yeast, landmark proteins deposited during the previous cell cycle determine the axis of polarity. These positional cues marking the future site of bud emergence are thought to recruit scaffold proteins (such as Bem1p), GTPases (Rsr1p, Cdc42p) and their regulators (Bud2p, Cdc24p…) (For review see [3]). Cdc42p is assumed to activate formins which in turn nucleate actin filaments that are specifically assembled into actin cables (for review see [4]). Thus, actin cable nucleation is thought to take place mainly at the site of polarity establishment. Actin cables serve as tracks for type V myosins-mediated polarized transport of secretory vesicles towards the site of bud emergence (for review [2]). Once delivered, the vesicles dock and fuse with the plasma membrane allowing polarized cell growth.

Even though the initial recruitment of Cdc42p can occur independently of the actin cytoskeleton [5]–[8], actin cables clearly contribute to maintain Cdc42p at the bud tip 5, 6. Furthermore, actin patch-mediated endocytosis was shown to disperse Cdc42p from the pre-bud site [6]. Thus, Cdc42p polarization at the presumptive bud site involves a dynamic and antagonistic interplay between distinct F-actin containing structures [9]–[11].

Budding yeast treated with latrunculin-A (Lat-A), a drug that prevents F-actin polymerization, or cells specifically lacking actin cables, have been shown to grow in an isotropic manner [8], [12], [13]. Therefore, the actin cytoskeleton seems not required for secretion *per se* but rather for the polarized delivery of secretory vesicles toward the site of growth. Intriguingly, in *Schizosaccharomyces pombe*, cells lacking the formin for3p do not display any detectable interphase actin cables and have depolarized actin patches, yet these cells are viable and exhibit some degree of polarized growth [14].

Whether the actin cytoskeleton is required for polarity establishment or is only essential to maintain the polarization of growth remains an unclear issue. This question is further overshadowed by the fact that in rapidly dividing yeast cells, the actin cytoskeleton is almost always polarized. Our recent discovery of actin bodies, a non polarized F-actin containing structure that is specific of yeast quiescent cells [15] prompted us to re-examine the requirement of F-actin containing structures for the initiation of polarized growth in *S. cerevisiae*.

## Results

### Polarized growth in the absence of actin cables

We have recently shown that after 7 days of growth in rich medium, yeast cells display a specific actin cytoskeleton organization that we have named actin bodies. Actin bodies are dense F-actin containing structures that are not polarized. Within minutes upon cell re-feeding, actin bodies disappear and depolarized actin patches and cables are concomitantly assembled. These structures then polarize toward the site of bud emergence and new budded cells with polarized actin cytoskeleton appear within two hours ([15] and Supplementary Figure S1A-C). As previously reported by others [16], [17], we observed here that haploid yeast cells exiting quiescence displayed a specific budding pattern, *i.e.*: the vast majority of daughter and mother cells emitted a new bud at the distal pole (Supplementary Figure S1D and data not shown). This budding pattern is consistent with the maintenance of long term distal polarity landmarks proteins in quiescent yeast cells [16].

**Figure 1:**
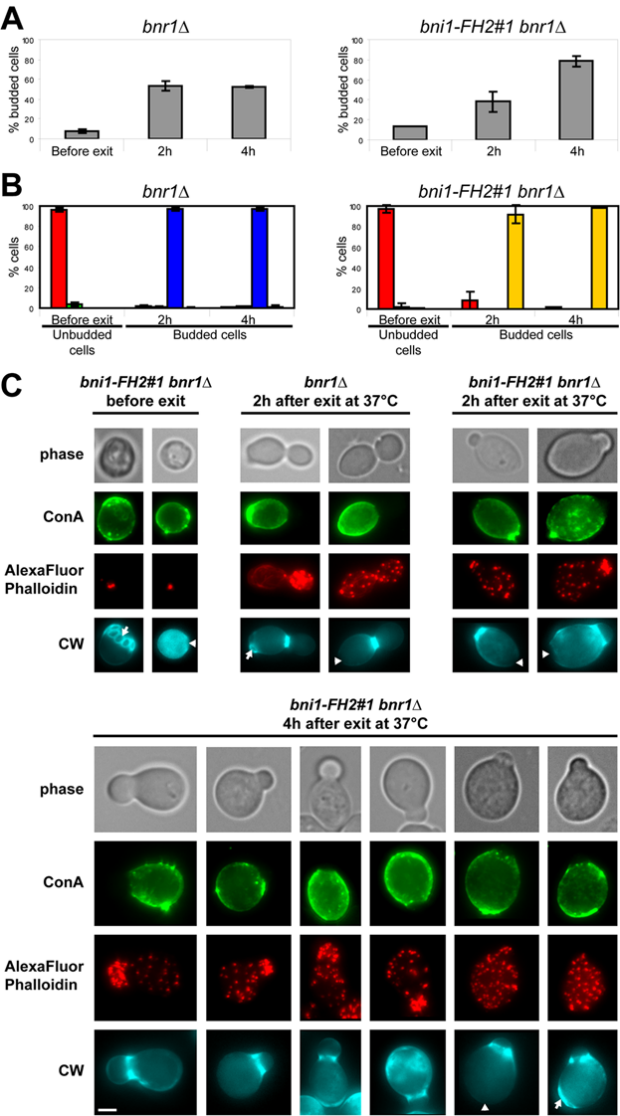
Bud emergence in the absence of actin cables. *bnr1Δ* and *bni1-FH2#1 bnr1Δ* cells were grown 7 days in YPDA medium at 25°C. Cells were incubated with Con-A-FITC for 1 h, then washed with “old” YPDA and shifted to 37°C for 30 min as described in material and methods. Cells were then re-fed with pre-warmed YPDA medium and grown at 37°C. (A) Percentage of budded cells in *bnr1Δ* and *bni1-FH2#1 bnr1Δ* cultures before and after exit from quiescence at 37°C, N>200 for each time point, 2 experiments – error bars show SD. (B) Actin cytoskeleton organization in *bnr1Δ* and *bni1-FH2#1 bnr1Δ* cells before and after exit from quiescence at 37°C. Red: Actin Bodies; green: depolarized actin patches and cables; blue: polarized actin patches and cables; yellow: no detectable actin cable and depolarized actin patches (N>200 for each time point, 2 experiments – error bars show SD). (C) Images of typical *bnr1Δ* and *bni1-FH2#1 bnr1Δ* cells. Arrows indicate bud scars, arrowheads indicate birth scars; CW: Calcofluor White. Bar 2 µm.

We first addressed the role of actin cables in the establishment and the maintenance of polarized growth upon exit from quiescence using a thermo-sensitive (ts) mutant strain conditional for formin function: *bni1-FH2#1 bnr1Δ*. When shifted to non-permissive temperature during exponential growth, these mutant cells specifically lose actin cables within a few minutes [18]. After 7 days of growth in rich medium at 25°C, the majority of *bni1-FH2#1 bnr1Δ* cells displayed actin bodies, indicative of a proper entry into quiescence (Figure 1, B and C). The stationary phase culture was pre-shifted at 37°C for 30 min to ensure the inactivation of Bni1-FH2#1p. The exit from quiescence was then triggered by transferring the cells into pre-warmed rich medium. As expected, 2 h after exit from quiescence at 37°C, *bni1-FH2#1 bnr1Δ* cells did not display any detectable actin cables (Figure 1, B and C), but strikingly, many cells with a new bud were observed. Based on the constriction between the mother and the “daughter”, we call these cells “budded cells”. Furthermore, the concanavalin A (Con-A) staining (Figure 1C) testified that buds were formed by *de novo* polarized growth. Although in the early time points, bud emergence was less efficient in the formin ts strain than in the *bnr1Δ* or in the WT control strains (Figure 1A and supplementary Figure S1), 4 h after exit from quiescence at 37°C, the percentage of budded cells was higher in the formin ts strain than in the control strains. Indeed, formin ts cells did not complete the cell cycle and remained budded whereas the control strains started another round of cell division. Importantly, in formin ts cells, just as in wild type cells, the new bud emerged at the distal pole (97 +/− 2% of daughter cells emitted a new bud at the distal pole, N>200, 2 experiments). This indicates that formin ts cells used long-term distal polarity landmarks upon exit from quiescence. Consistently, it has recently been reported that a formin conditional mutant was able to initiate bud formation upon release from α-factor arrest [19]. We further confirmed our observations using a tropomyosin ts strain. Tropomyosins are required for actin cable maintenance [13]. As formin ts cells, upon exit from quiescence at restrictive temperature, tropomyosin ts cells were able to form a new bud at the distal pole (Supplementary Figure S2). From those experiments, we conclude that upon exit from quiescence, actin cables do not appear to be required for bud emergence at the distal pole. Additionally, these results show that actin cables are apparently not required to sustain the primary steps of polarized growth, since cells in which formins or tropomyosins were inactivated could grow a bud of significant size. However, in both mutants, the bud necks were widened and mother cells were abnormally round (see Figure 1C and Supplementary Figure S2) revealing an impaired overall long-term maintenance of cell polarity (see discussion).

### Polarized growth in the absence of actin patch-mediated endocytosis

**Figure 2:**
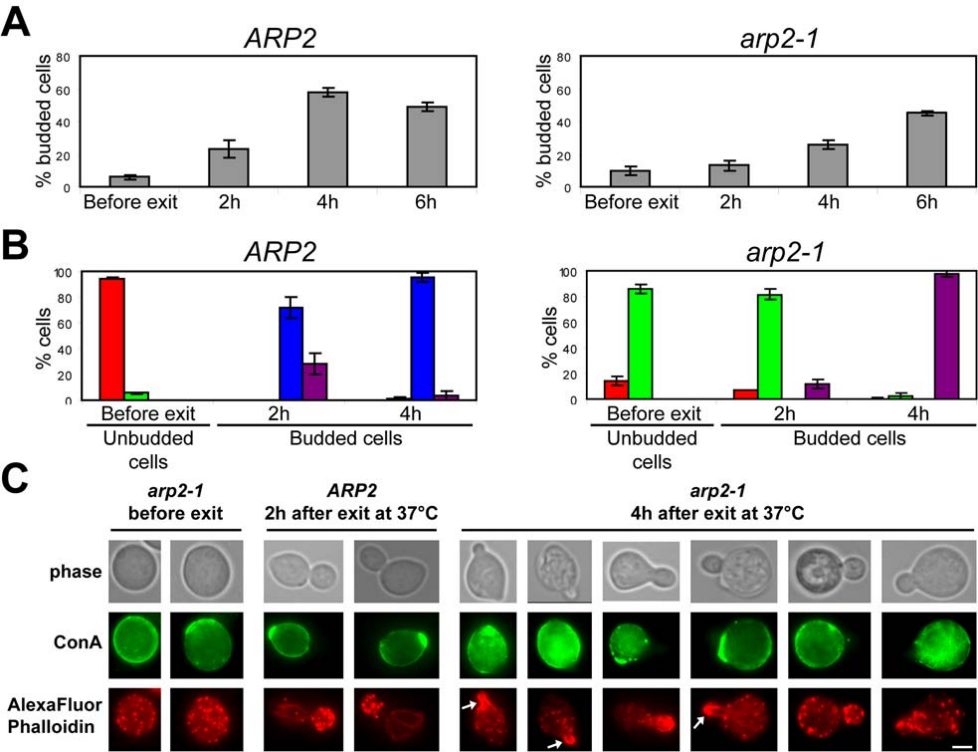
Bud emergence in the absence of functional actin patches. *ARP2* and *arp2-1* cells were grown 7 days in YPDA medium at 25°C. Cells were incubated with Con-A-FITC for 1 h, then washed with “old” YPDA and shifted to 37°C for 1 h as described in material and methods. Cells were then re-fed with pre-warmed YPDA medium and grown at 37°C. (A) Percentage of budded cells in *ARP2* and *arp2-1* cultures before and after exit from quiescence at 37°C, N>200 for each time point, 2 experiments – error bars show SD. (B) Actin cytoskeleton organization in *ARP2* and *arp2-1* cells before and after exit from quiescence at 37°C. Red: Actin Bodies; green: depolarized actin patches and cables; blue: polarized actin patches and cables; purple: abnormal actin cables and depolarized actin patches (N>200 for each time point, 2 experiments – error bars show SD). (C) Image of typical *ARP2* and *arp2-1* cells. Arrows indicate abnormally big actin cable-like structures. Bar 2 µm.

Actin patches are required for endocytosis. The polymerization of branched actin filaments that mediate the internalization of endocytic vesicles is nucleated by the Arp2/3 complex and is regulated by a large set of proteins (For review see [20]). To decipher the role of actin patch-mediated endocytosis in bud emergence, we used the temperature sensitive *arp2-1* strain [21] in which the Arp2/3 complex is impaired for actin nucleation [22]. At non permissive temperature, exponentially growing *arp2-1* cells are defective for the internalization step of endocytosis [23]. *Arp2-1* cells were grown 7 days at 25°C. Surprisingly, even at permissive temperature, these cells did not display actin bodies, but rather depolarized actin patches and an abnormally dense network of non-polarized actin cables (Figure 2C and data not shown). This indicated that an impairment of Arp2/3 complex activity prevented actin bodies formation. To inactivate the Arp2/3 complex, the 7 days old *arp2-1* cell culture was pre-incubated 1 h at 37°C. Cells were then re-fed with pre-warmed rich medium and grown at 37°C. As shown in Figure 2A, upon exit from quiescence, even at non permissive temperature, polarized growth can occur in *arp2-1* cells, although less efficiently than the WT control. As expected, in these cells, actin patches monitored with an Abp1p-3xGFP were defective for non linear movement that occurs upon endosome internalization [24] (data not shown). Additionally, F-actin staining using AlexaFluor phalloidin revealed that cells undergoing *de novo* polarized growth display a typical Arp2/3 complex-defective actin cytoskeleton, i. e. depolarized patches and abnormally big actin cable-like structures into the daughter cell (Figure 2, B and C and supplementary Figure S3; [25]). After 4 h at 37°C, *arp2-1* cells died and lysed [21]. This probably accounts for the low percentage of newly budded cells in this mutant. Finally, the budding pattern of the newly budded cells could not be investigated since *arp2-1* mutant cells display a random budding pattern even at permissive temperature [21]. These results indicate that actin patches function is not strictly required for bud emergence and the first steps of polarized growth upon exit from quiescence. However, in the *arp2-1* mutant, the emerging buds are rather small and misshaped, confirming that endocytosis, like actin cables-mediated polarized transport, is required to sustain normal polarized growth.

### Polarized growth in the absence of F-actin containing structures

**Figure 3:**
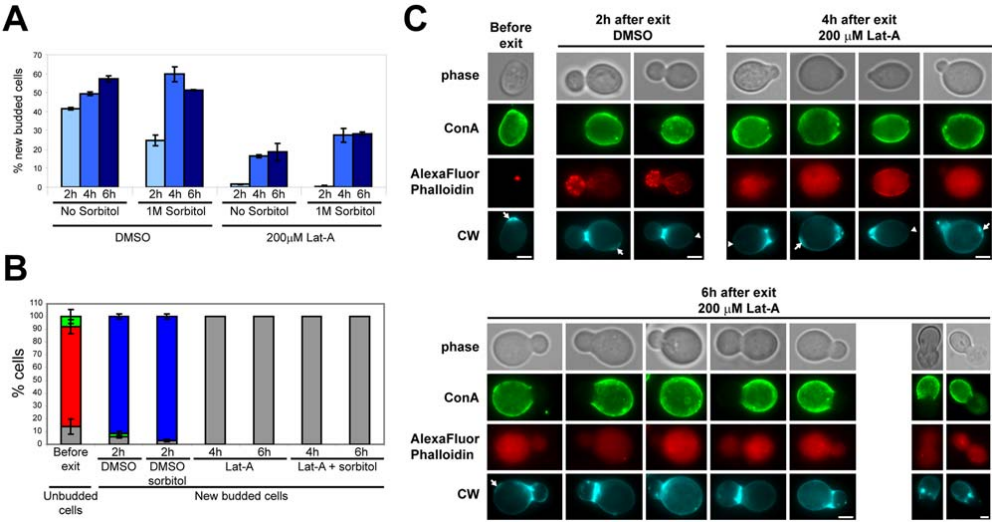
Bud emergence in the absence of F-actin. Wild type cells were grown 7 days in YPDA medium at 30°C. Cells were incubated with Con-A-FITC for 1 h and then washed with “old” YPDA. Lat-A (200 µM final concentration) or DMSO were added and cells were further incubated at 30°C for 30 min. Cells were then re-fed with YPDA medium (with or without 1 M sorbitol) containing either 200 µM Lat-A or DMSO and grown at 30°C as described in material and methods. (A) Percentage of new budded cells after exit from quiescence at 30°C, N>200 for each time point, 2 experiments – error bars show SD. (B) Actin cytoskeleton organization in unbudded cells before exit from quiescence or in new budded cells after exit from quiescence at 30°C in YPDA medium containing either DMSO, DMSO and 1 M sorbitol, 200 µM Lat-A or 200 µM Lat-A and 1 M sorbitol. Red: Actin Bodies; green: depolarized actin cytoskeleton; blue: polarized actin cytoskeleton; grey: no detectable F-actin containing structures (N>200 for each time point, 2 experiments – error bars show SD). (C) Images of typical treated and untreated cells. The upper left panel display a typical wild type cell pre-incubated 30 min with Lat-A before re-feeding and the lower right panel, examples of Lat-A treated wild type cells 6 h after re-feeding with a collapsed new bud. Arrows indicate bud scar, arrowhead indicate birth scars; CW: Calcofluor White. Bar 2 µm. Of note, for AlexaFluor Phalloidin images, the maximum intensity is about 20 times lower for Lat-A treated cells than for untreated cells and was therefore greatly enhanced in the figure to document the absence of F-actin structure.

Since neither actin cables nor actin patches alone are apparently required for bud emergence upon exit from quiescence, we asked whether depleting all F-actin containing structures would allow polarized growth. Latrunculin-A (Lat-A) prevents actin polymerization by interacting with actin monomers and results in the rapid disassembly of dynamic F-actin structures such as cables and patches [8]. Yet, because the turn over of actin filaments embedded into actin bodies is slow, these structures remained detectable even after a 2 h treatment with 200 µM Lat-A. However, upon cells re-feeding in the presence of 200 µM Lat-A, actin bodies promptly disappeared and no Abp1-3xGFP-containing structures (i. e. actin patches) could be observed [15]. Wild type cells grown 7 days at 30°C were pre-treated for 30 min with 200 µM of Lat-A and then re-fed in 200 µM of Lat-A-containing rich medium. As shown in Figure 3, A and C, 4 h after re-feeding, a small but significant number of Lat-A treated cells could undergo *de novo* polarized growth. The number of new budded cells did not increase with time because the newly formed buds were fragile and lysed (Figure 3C, lower right panel). Indeed, triggering exit from quiescence in rich medium containing 200 µM of Lat-A and 1 M sorbitol significantly increased the number of new budded cells (Figure 3A). Importantly, new buds emerged at the distal pole (98% of daughter cells and 87% of mother cells displayed *de novo* polarized growth at the distal pole). We have verified that Bem1p, a scaffold protein important for polarity establishment, is polarized at the tip of the new buds (Supplementary Figure S4A; [8], [26]). As expected, due to the Lat-A treatment, in cells with a new bud, no F-actin-containing structures could be detected by AlexaFluor phalloidin staining (Figure 3, B and C). We confirmed that Lat-A treated cells exiting quiescence were not displaying detectable actin cables using cells expressing Abp140p-GFP (Supplementary Figure S4B). Finally, to re-enforce our findings, we used jasplakinolide, a drug that stabilizes F-actin containing structures and causes a rapid accumulation of large actin clumps in exponentially growing yeast cells [27]. After a 30 min pre-treatment with 10 µM of jasplakinolide, quiescent cells were released in rich medium containing 10 µM of jasplakinolide. Following 2 and 4 h in the presence of the drug, some cells with typical jasplakinolide-induced actin aggregates that have undergone *de novo* polarized growth could be observed (Supplementary Figure S4C). These newly emerged buds remained small and cells lysed rapidly and even more dramatically than in the Lat-A experiment. These results therefore demonstrate that upon exit from quiescence, F-actin-containing structures are neither strictly required for establishing polarity nor for sustaining early steps of polarized growth.

### Polarized secretion in the absence of F-Actin

**Figure 4:**
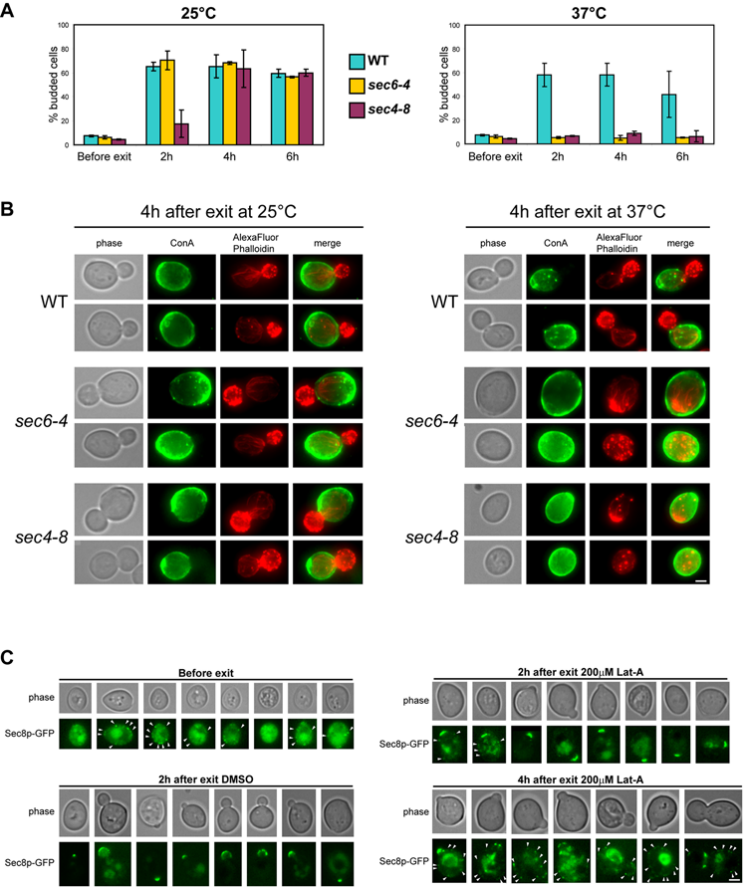
Bud emergence requires a functional exocyst. (A) *sec4-8*, *sec6-4* and isogenic wild type cells were grown 7 days in YPDA medium at 25°C. Cells were incubated with Con-A-FITC then washed with “old” YPDA and cells were shift to 37°C for 1 h. Cells were then re-fed with pre-warmed YPDA medium and grown either at 25°C or at 37°C as described in material and methods. The percentage of budded cells in wild type (blue), *sec4-8* (purple) and *sec6-4* (yellow) is indicated before and after exit from quiescence at 25°C or 37°C, N>200 for each time point, 2 experiments – error bars show SD. (B) Image of typical *WT*, *sec6-4 and sec4-8* cells, 4 h after exiting quiescence at 25°C (left) and 37°C (right). (C) Localization of Sec8p-GFP before and after exit from quiescence at 30°C in the absence or in the presence of 200 µM Lat-A. Arrowheads point at Sec8p-GFP dots at the cell periphery. Bar 2 µm.

Our data demonstrate that in cells where polarity landmarks are present, the actin cytoskeleton is not required for the first steps of polarized growth upon exit from quiescence. In budding yeast, microtubules seem not to be involved in polarized growth [1], [2] and we have verified that cells exiting quiescence in the presence of both Lat-A and nocodazole, a drug that affects microtubule polymerization, were able to form new buds (data not shown). In contrast, results presented in Figure 4A and B show that functional secretion machinery is strictly required for polarized growth upon exit from quiescence, as previously demonstrated in rapidly dividing cells [28]. Indeed, thermo-sensitive mutants for the exocyst function were unable to emerge a new bud upon exit from quiescence. This prompted us to localize the secretion machinery in cells undergoing polarized growth in the absence of F-actin containing structures upon exit from quiescence. As shown in Figure 4C, in cells exiting from quiescence in the presence of Lat-A, Sec8p-GFP, a component of the exocyst, could be detected as discrete dots all around the cell periphery, with an enrichment at the site of emerging bud or at tip of the new small buds (Figure 4C). However, this enrichment was no longer visible as incubation time in the presence of Lat-A increased and as new buds grew (Figure 4C, lower panel). The same results were obtained for Sec5p-GFP (data not shown). These observations, which strongly suggest that under these conditions growth is not restricted to the bud, are in good agreement with the fact that in the absence of F-actin, mother cells are abnormally rounded. However our results are in contradiction with those previously reported by Ayscough *et al*, a study in which Sec4p and Sec8p were not polarized in cells exiting from early stationary phase in the presence of Lat-A [8]. We do not know what could account for these discrepancies but since, in the Ayscough study, those proteins were detected by immuno-fluorescence, we can speculate that the slight enrichment of the signal at the polarization site was not detectable using this technique. Alternatively, it could be that fragile newly budded cells were lost during the chemical treatments used for immuno-detection. Consistently with our results, in a recent study, France *et al* have shown that the exocyst component Sec15p was able to polarize in more than 70% of the cells upon exit from quiescence in the presence of Lat-A [29]. In this study, it was also shown that Sec8p can be detected as polarized foci in more than 20% of cells exiting quiescence in the presence of Lat-A.

## Discussion

Here we have shown that in yeast, F-actin containing structures are not required for the first steps of polarized growth and bud emergence upon exit from quiescence. Our results contrast with previous studies where it has been shown that Lat-A treatment in vegetative cells prevents new bud formation (See for example [12]). More intriguingly, Ayscough *et al* and Bi *et al* have previously observed that Lat-A treatment of early stationary phase cells inhibits bud emergence [8], [30]. We have shown that 7 days old quiescent cells are capable to re-assemble depolarized patches and cables within few minutes after release in fresh medium [15]. In the earlier studies, stationary phase unbudded cells were sorted by differential centrifugation in rich YPD. It is thus very likely that these cells had already exited quiescence and therefore have already assembled cables and patches before Lat-A addition. These cells might therefore behave more like actively growing cells rather than *bona fide* quiescent cells. In our experimental design, more than 90% of the quiescent cells show non-polarized actin structures (actin bodies) upon Lat-A addition. Furthermore the Con-A labeling testifies that the observed buds are indeed new buds. Several differences between vegetative and 7 days old quiescent haploid cells could account for these discrepancies, for example the site of bud emergence and therefore the active polarity landmarks are different; moreover *de novo* protein synthesis is required for polarity establishment upon exit from quiescence [15] suggesting that in quiescence key polarity regulators are missing. Whatever the differences between quiescent and actively dividing cells, this work demonstrates that F-actin is not strictly required for early steps of polarized growth.

**Figure 5:**
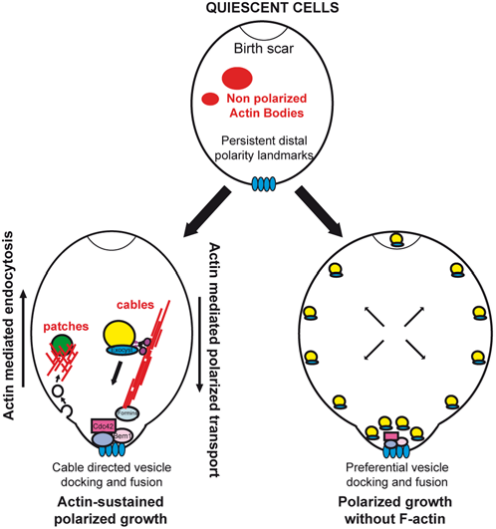
Model for polarized growth maintenance in the presence or in the absence of F-Actin containing structures. See text for details.

We propose a model in which, upon exit from quiescence in the absence of F-actin, secretory vesicles emanate in all directions, but preferentially dock and fuse at the distal pole allowing bud emergence where long term polarity landmarks are localized (See Figure 5). Upon exit from quiescence, these tags are sufficient to recruit Cdc42p independently of polymerized actin, which is consistent with the fact that Cdc42p initial polarization can occur in the absence of F-actin [5]–[8]. Cdc42p could then interact with Sec3p [31], [32] a protein known to polarize independently of F-actin [33]–[35] through its interaction with Cdc42p [31], [32], [35]. Sec3p would therefore be able to facilitate the local tethering and fusion of exocyst coated secretory vesicles. Other polarity factors like Bem1p or protein involved in tethering and fusion of secretory vesicles such as Exo70p have also been shown to polarize in the absence of F-actin [8], [26], [35] and could therefore be crucial in this process. This cascade of events triggers the initiation of polarized growth and bud emergence upon exit from quiescence. However, in the absence of F-actin, this local “activation” would not be sustained and hence would not be sufficient to maintain a normal polarized secretion. Therefore, in quiescent cells in which polarity is established by polarity landmarks, F-actin containing structures are not required for polarized growth initiation, but rather to maintain long term polarized bud growth.

The specific impairment of actin patch-mediated endocytosis through the *arp2-1* mutation led to rapid cell death. Therefore, it is hard to conclude about a specific role for actin patches in polarized growth maintenance. Because a large variety of mutants display depolarized actin patches without having critical defects in polarized growth [36], polarization of endocytosis *per se* is clearly not crucial for polarized growth. Thus, the function of actin patches rather than their polarized localization seems critical for polarized growth. Since, Lat-A or jasplakinolide treated cells, but not actin cables-depleted cells, had a strong tendency to lyse upon exit from quiescence, it is reasonable to speculate that actin patch-mediated endocytosis is indispensable for the formation and/or the maintenance of the plasma membrane and the cell wall and may also affect the cell wall integrity pathway.

In the absence of F-actin containing structures, cells exiting quiescence display an abnormally wide bud neck. This observation has been previously reported for *bni1Δ* and polarisome defective mutants [37] and is also observed here in cells where formins or tropomyosins are inactivated (Figure 1 and Supplementary figure S2). Therefore, this defect seems to be specific of actin cable-defective cells. Whether the widening of the bud neck is due to an improper septin assembly or to a defect in actin ring formation await for further investigation. While polarized docking and fusion of secretory vesicles is mandatory for bud emergence, our data show that this process can occur in the absence of polarized actin tracks. Consistently, previous studies have suggested that actin cables polarization, which is abolished in a *bni1Δ bnr1Δ* mutant expressing a non polarized activated form of Bni1p, was not required for cell proliferation [38]. We propose that upon bud emergence, actin cables greatly enhance polarized growth efficiency by directing the transport of secretory vesicles. Although not mandatory for bud emergence and the early steps of bud growth, formins and tropomyosins are clearly required for cell cycle completion, possibly because of their crucial function in cytokinesis.

## Materials and Methods

### Yeast strains, growth conditions and specific staining

The *S. cerevisiae* wild type strain used in this study is BY4741 available from Euroscarf (Frankfurt, Germany). Temperature sensitive mutants used in this study have been described previously: *bnr1Δ* and *bni1-FH2#1 bnr1Δ* [18], *tpm2Δ* and *tpm1-1 tpm2Δ* strains [13], *arp2-1* strain and its congenic WT strain [39], *sec4-8*, *sec6-4* and the congenic WT [40]. The Abp1-3xGFP (P3006) and Sec8p-GFP constructs have been previously described in [15] and [41], respectively. Sec8p-GFP imaging was done in live cells. The YPDA medium was described previously in [15]. In all the experiments carried out in this study, yeast cells were grown 7 days in liquid YPDA at 25°C (ts mutants) or 30°C (Lat-A experiment) in 100 ml Erlenmeyer flasks with 220 rpm shaking. Cells were then concentrated in the same medium by low speed centrifugation. The remaining supernatant, i e the “old” YPDA medium, was filtered to remove non-pelleted cells. Concanavalin A-FITC (Sigma-Aldrich, St Louis, MO) was added to the concentrated cells to a final concentration of 0.2 mg/ml. Cells were then incubated 1 h at 25°C then washed twice with old filtered YPDA medium. In case of temperature shift, cells were re-suspended in old filtered YPDA medium and pre-shifted for 30 min or 1 h at 37°C in a water bath. Cells were then re-fed with pre-warmed YPDA (37°C) at an OD_600 nm_ of 0.6∼0.8 and grown in as liquid culture in a 37°C water bath. At the various time points after re-feeding, aliquots of cells were immediately fixed with formaldehyde (3.7% final) for at least one hour at the culture temperature. Cells were then stained with AlexaFluor568-phalloidin (Invitrogen, Carlsbad, CA) as described in [18]. Calcofluor white (Sigma-Aldrich, St Louis, MO) was added before the last wash to the final concentration of 2 µg/ml. Cells were then incubated 5 min at room temperature, washed with PBS and re-suspended in mounting solution (PBS, glycerol 50%, paraphenylenediamide 0.05%) and imaged. Lat-A was a very generous gift of B. Goode. For Lat-A experiments, cells were grown 7 days in YPDA at 30°C, and stained with Con-A as described. Before re-feeding, cells were incubated 30 min in old YPDA medium containing 200 µM of Lat-A or DMSO, then re-fed in YPDA medium containing 200 µM Lat-A with or without 1 M sorbitol.

### Epifluorescence Microscopy

Cells were observed in a fully automated Zeiss 200 M inverted microscope (Carl Zeiss, Thornwood, NY) equipped with an MS-2000 stage (Applied Scientific Instrumentation, Eugene, OR), a Lambda LS 175 W xenon light source (Sutter Instrument, Novato, CA), a 100× 1.4 numerical aperture Plan-Apochromat objective, and a five positions filter turret. Filter cubes were as follows: for Alexa-phalloidin 568: Cy3 (Ex: HQ535/50 – Em: HQ610/75 – BS: Q565lp), for live cells GFP: Endow GFP longpass (Ex: HQ470/40 – Em: HQ500lp – BS: Q495lp), for Con-A-FITC Narrowband HQ FITC (Ex: HQ487/25 – Em: HQ535/40 – BS: Q505lp) and for Calcofluor White DAPI/Hoechst/AMCA (Ex: D360/40 – Em: D460-50 – BS: 400dclp) (Chroma Technology, Rockingham, VT). Images were acquired using a CoolSnap HQ camera (Roper Scientific, Tucson, AZ). The microscope, camera, and shutters (Uniblitz, Rochester, NY) were controlled by SlideBook software (Intelligent Imaging Innovations, Denver, CO). The objective heater was from Bioptechs (Butler, PA). Images are, unless specified, maximal projection of Z-stacks performed using a 0.2 or 0.3 µm step.

## Supporting Information

Figure S1 (A) to (D) Wild type cells were grown 7 days in YPDA medium at 25°C. Cells were incubated with Con-A-FITC then washed with “old” YPDA and then shifted to 25°C or 37°C for 30 min. Cells were then re-fed either with YPDA medium and grown at 25°C or with pre-warm YPDA medium and grown at 37°C as described in material and methods. (A) Percentage of budded cells in wild type cultures before and after exit from quiescence at 25°C and 37°C. (N≥200 for each time point, 2 experiments - error bars show SD). (B) Actin cytoskeleton organization in wild type cell at 25°C or 37°C. Red: Actin Bodies; green: depolarized actin patches and cables; blue: polarized actin patches and cables (N≥200 for each time point, 2 experiments - error bars show SD). (C) Image of typical wild type cells; left panel: before re-feeding at 25°C; 2 h after re-feeding at 37°C. Arrows indicate bud scar, arrowhead indicate birth scars; CW: Calcofluor White. Bar 2 µm. (D) Budding pattern of wild type daughter cells 2 h after exit from quiescence at 30°C (N≥200 for each time point, 2 experiments). (E) Percentage of budded cells in *bnr1Δ* and *bni1-FH2#1 bnr1Δ* cultures before and after exit from quiescence at 25°C (N≥200 for each time point, 2 experiments - error bars show SD). (F) Actin cytoskeleton organization in *bnr1Δ* and *bni1-FH2#1 bnr1Δ* cells before and after exit from quiescence at 25°C. Red: Actin Bodies; green: depolarized actin patches and cables; blue: polarized actin patches and cables; yellow: no detectable actin cable and depolarized actin patches (N≥200 for each time point, 2 experiments - error bars show SD).
(9.80 MB TIF)

Figure S2 *tpm2Δ* and *tpm1-1 tpm2Δ* cells were grown 7 days in YPDA medium at 25°C. Cells were then incubated with Con-A-FITC for 1 h then washed with “old” YPDA as described in materiel and methods. Cells were then shift to 25°C or 37°C for 30 min and re-fed either with YPDA medium and grown at 25°C or with pre-warmed YPDA medium and grown at 37°C. (A) Percentage of budded cells in *tpm2Δ* and *tpm1-1 tpm2Δ* cultures before and after exit from quiescence at 25°C or 37°C (N≥200 for each time point, 2 experiments - error bars show SD). (B) Actin cytoskeleton organization in *tpm2Δ* and *tpm1-1 tpm2Δ* cells before and after exit from quiescence at 25°C or 37°C. Red: Actin Bodies; green: depolarized actin patches and cables; blue: polarized actin patches and cables; yellow: no detectable actin cable and depolarized actin patches (N≥200 for each time point, 2 experiments - error bars show SD). (C) Images of typical *tpm2Δ* and *tpm1-1 tpm2Δ* cells. Left panel: *tpm1-1 tpm2Δ* cell before re-feeding at 25°C; middle panel: *tpm2Δ* cell 2 h after re-feeding at 37°C; *tpm1-1 tpm2Δ* cells 4 h after re-feeding at 37°C. CW: Calcofluor White; Bar 2 µm. (D) Budding pattern of *tpm1-1 tpm2Δ* cells 4 h after exit from quiescence at 30°C (N≥100 for each time point, 2 experiments).
(8.73 MB TIF)

Figure S3 (A) Percentage of budded cells in *ARP2* and *arp2-1* cultures before and after exit from quiescence at 25°C. For details see materiel and methods (N≥200 for each time point, 2 experiments - error bars show SD). (B) Actin cytoskeleton organization in *ARP2* and *arp2-1* cells before and after exit from quiescence at 25°C. Red: Actin Bodies; green: depolarized actin patches and cables; blue: polarized actin patches and cables; purple: abnormal actin cables and depolarized actin patches (N≥200 for each time point, 2 experiments - error bars show SD).
(9.66 MB TIF)

Figure S4 (A) Localization of Bem1p-3xGFP in wild type cells exiting from quiescence. Three copies of GFP were integrated at the BEM1 locus. This fusion protein is functional since it can be expressed in a *bni1Δ* or *rsr1Δ* without affecting their growth. Details of the construct are available upon request. Wild type cells expressing Bem1p-3xGFP were grown 7 days at 30°C, treated for 30 min with 200 µM Lat-A or DMSO. Cells were then re-fed with YPDA medium containing either 200 µM Lat-A or DMSO and grown at 30°C as described in material and methods. Bar 2 µm. Histograms display the percentage of cells with polarized Bem1p-3xGFP (red), depolarized Bem1p-3xGFP (green), Bem1p-3xGFP slightly polarized in a diffuse crescent shape manner (blue) or Bem1p-3xGFP not detected (grey). N≥200 for each time point, 2 experiments - error bars show SD. (B) Localization of Abp140p-GFP in wild type cells exiting quiescence in the presence of Lat-A. Wild type cells expressing Abp140p fused to GFP (Invitrogen, Carlsbad, CA) were grown 7 days at 30°C, treated for 30 min with 200 µM Lat-A or DMSO. Cells were then re-fed with YPDA medium containing either 200 µM Lat-A or DMSO and grown at 30°C as described in material and methods. Bar 2 µm. Histograms display the percentage of cells with detectable Abp140p-GFP decorated actin structures 2 or 4 h after exit from quiescence in the absence or in the presence of 200 µM Lat-A (N≥100 for each time point, 2 experiments - error bars show SD). (C) Cells exiting quiescence in the presence of jasplakinolide. Jasplakinolide sensitive strain (*snq2Δ pdr5Δ erg6Δ* see (Ayscough, 2000) expressing Abp1p-3xGFP from the endogenous locus (Sagot et al, 2006) were grown 7 days at 30°C. Cells were then pre-treated with 10 µM jasplakinolide or DMSO and then re-fed in YPDA medium containing 10 µM jasplakinolide or DMSO. Left panel: *snq2Δ pdr5Δ erg6Δ ABP1-3xGFP* after 7 days of growth at 30°C. Of note, because of the erg6 deletion, *snq2Δ pdr5Δ erg6Δ ABP1-3xGFP* cells do not display actin bodies when grown to stationary phase (I.S. unpublished result). Middle panel: DMSO treated cells 4 h after re-feeding. In our hands, exponentially growing *snq2Δ pdr5Δ erg6Δ ABP1-3xGFP* cells display a slightly depolarized actin cytoskeleton, as revealed in DMSO treated cells, 4 h after exit from quiescence. Right panel: jasplakinolide treated cells 4 h after re-feeding (N≥200, 2 experiments), bar 2 µm.
(10.06 MB TIF)
