# Supplementary figures and images for: Polarized Growth in the Absence of F-Actin in Saccharomyces cerevisiae Exiting Quiescence
Source: PLoS One. 2008 Jul 2;3(7):e2556. doi: 10.1371/journal.pone.0002556 (PMC2440520; doi:10.1371/journal.pone.0002556)

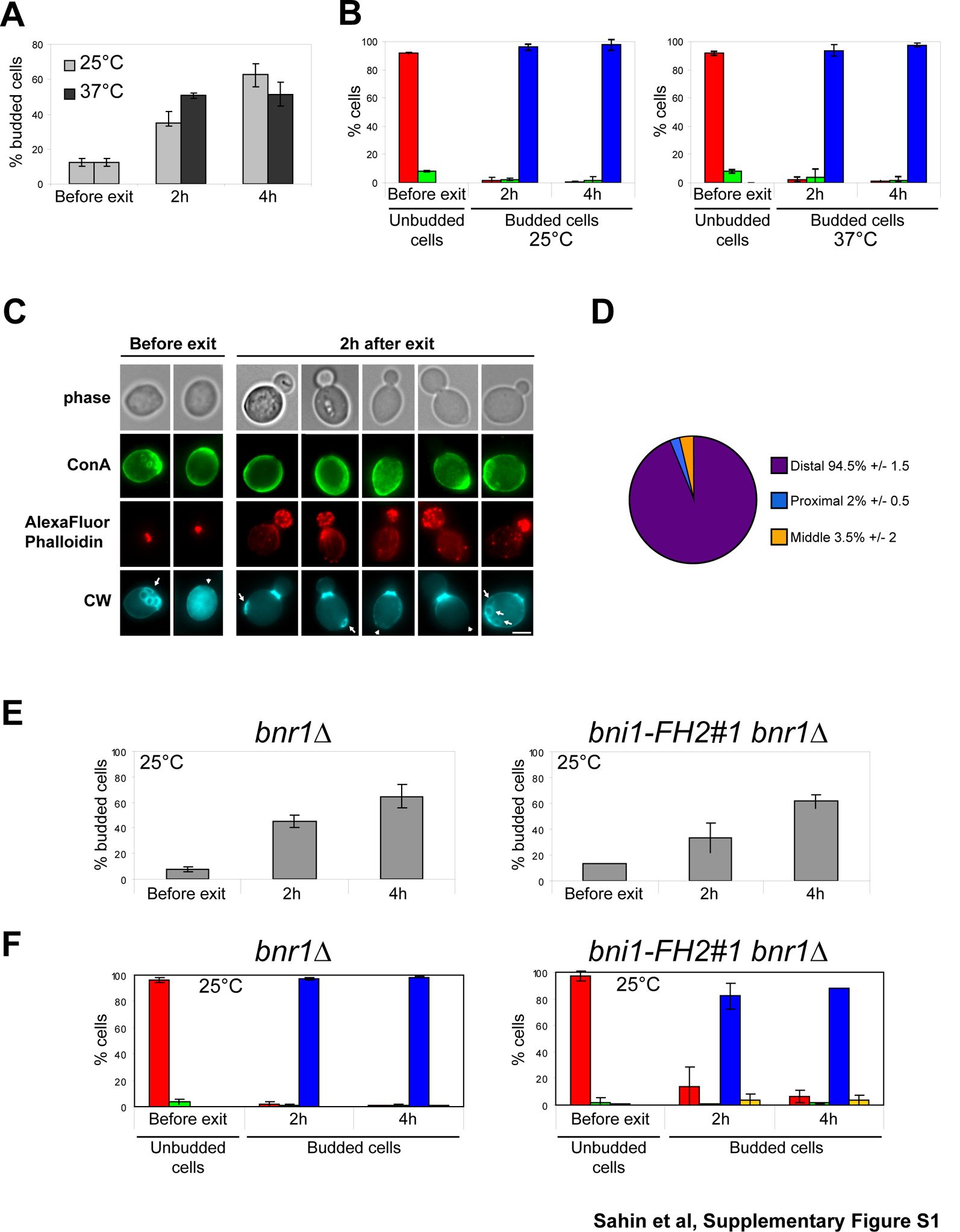

Supplement: Figure S1 — (A) to (D) Wild type cells were grown 7 days in YPDA medium at 25°C. Cells were incubated with Con-A-FITC then washed with “old” YPDA and then shifted to 25°C or 37°C for 30 min. Cells were then re-fed either with YPDA medium and grown at 25°C or with pre-warm YPDA medium and grown at 37°C as described in material and methods. (A) Percentage of budded cells in wild type cultures before and after exit from quiescence at 25°C and 37°C. (N≥200 for each time point, 2 experiments - error bars show SD). (B) Actin cytoskeleton organization in wild type cell at 25°C or 37°C. Red: Actin Bodies; green: depolarized actin patches and cables; blue: polarized actin patches and cables (N≥200 for each time point, 2 experiments - error bars show SD). (C) Image of typical wild type cells; left panel: before re-feeding at 25°C; 2 h after re-feeding at 37°C. Arrows indicate bud scar, arrowhead indicate birth scars; CW: Calcofluor White. Bar 2 µm. (D) Budding pattern of wild type daughter cells 2 h after exit from quiescence at 30°C (N≥200 for each time point, 2 experiments). (E) Percentage of budded cells in bnr1Δ and bni1-FH2#1 bnr1Δ cultures before and after exit from quiescence at 25°C (N≥200 for each time point, 2 experiments - error bars show SD). (F) Actin cytoskeleton organization in bnr1Δ and bni1-FH2#1 bnr1Δ cells before and after exit from quiescence at 25°C. Red: Actin Bodies; green: depolarized actin patches and cables; blue: polarized actin patches and cables; yellow: no detectable actin cable and depolarized actin patches (N≥200 for each time point, 2 experiments - error bars show SD). (9.80 MB TIF) [file pone.0002556.s001.tif]

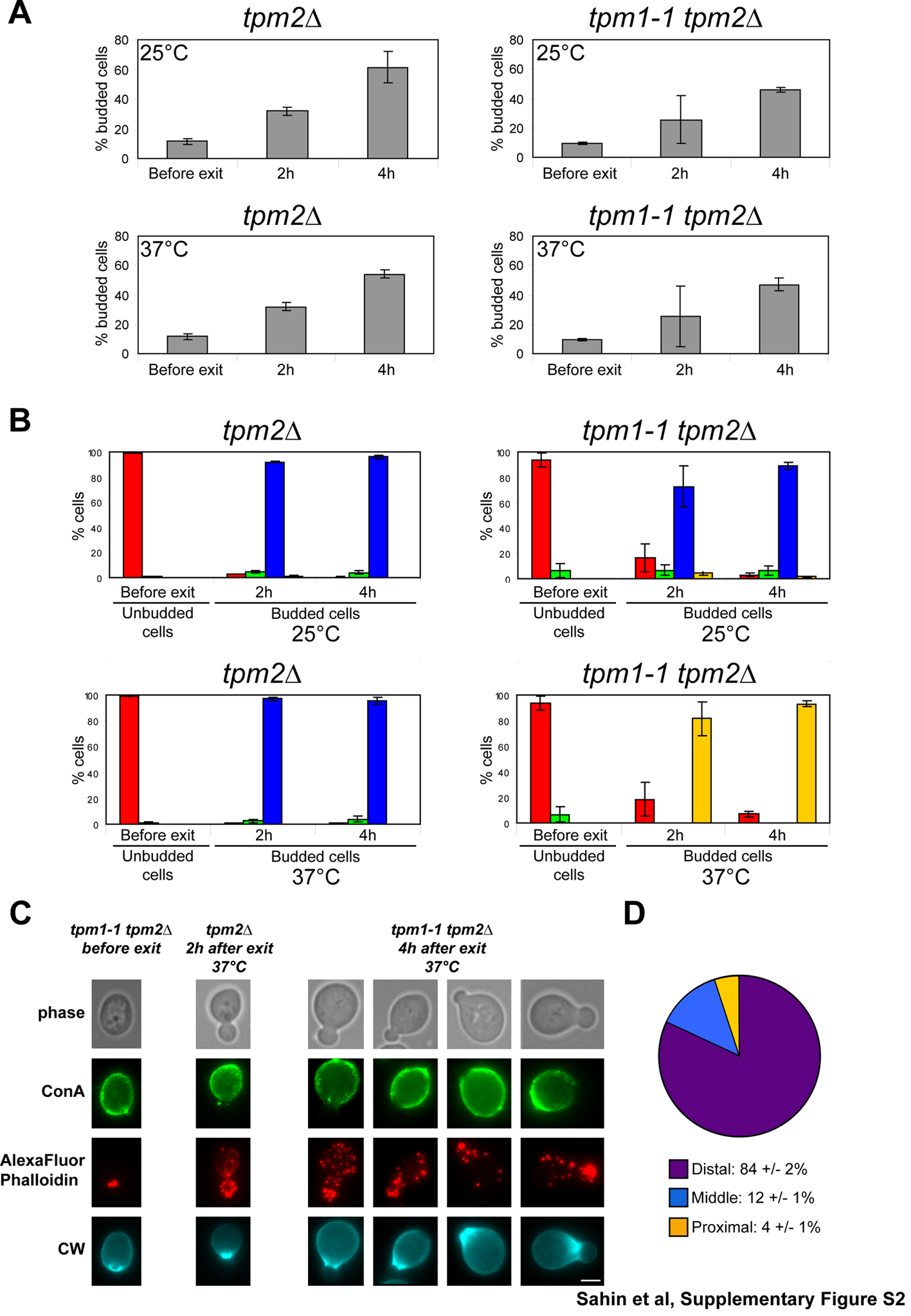

Supplement: Figure S2 — tpm2Δ and tpm1-1 tpm2Δ cells were grown 7 days in YPDA medium at 25°C. Cells were then incubated with Con-A-FITC for 1 h then washed with “old” YPDA as described in materiel and methods. Cells were then shift to 25°C or 37°C for 30 min and re-fed either with YPDA medium and grown at 25°C or with pre-warmed YPDA medium and grown at 37°C. (A) Percentage of budded cells in tpm2Δ and tpm1-1 tpm2Δ cultures before and after exit from quiescence at 25°C or 37°C (N≥200 for each time point, 2 experiments - error bars show SD). (B) Actin cytoskeleton organization in tpm2Δ and tpm1-1 tpm2Δ cells before and after exit from quiescence at 25°C or 37°C. Red: Actin Bodies; green: depolarized actin patches and cables; blue: polarized actin patches and cables; yellow: no detectable actin cable and depolarized actin patches (N≥200 for each time point, 2 experiments - error bars show SD). (C) Images of typical tpm2Δ and tpm1-1 tpm2Δ cells. Left panel: tpm1-1 tpm2Δ cell before re-feeding at 25°C; middle panel: tpm2Δ cell 2 h after re-feeding at 37°C; tpm1-1 tpm2Δ cells 4 h after re-feeding at 37°C. CW: Calcofluor White; Bar 2 µm. (D) Budding pattern of tpm1-1 tpm2Δ cells 4 h after exit from quiescence at 30°C (N≥100 for each time point, 2 experiments). (8.73 MB TIF) [file pone.0002556.s002.tif]

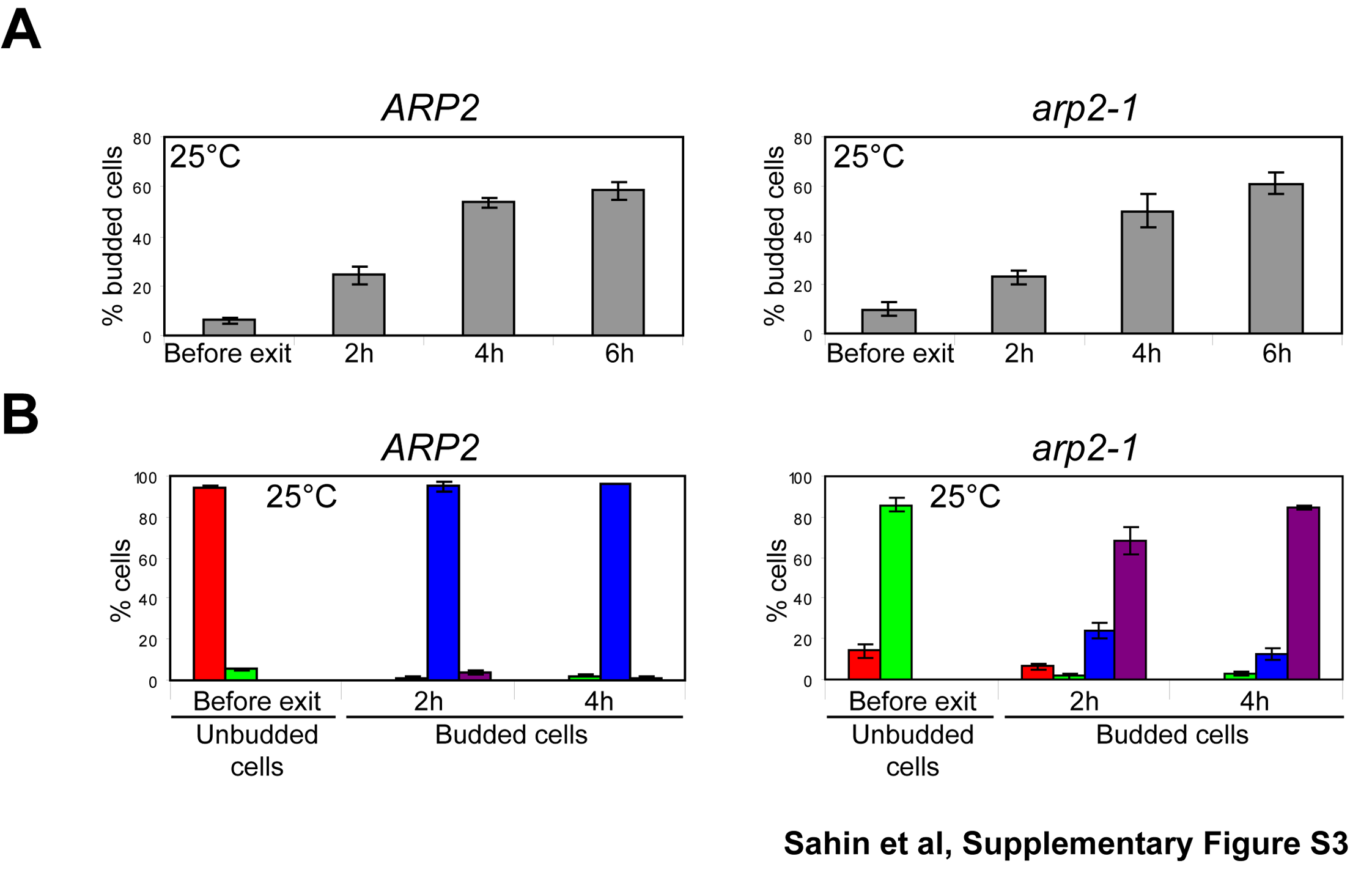

Supplement: Figure S3 — (A) Percentage of budded cells in ARP2 and arp2-1 cultures before and after exit from quiescence at 25°C. For details see materiel and methods (N≥200 for each time point, 2 experiments - error bars show SD). (B) Actin cytoskeleton organization in ARP2 and arp2-1 cells before and after exit from quiescence at 25°C. Red: Actin Bodies; green: depolarized actin patches and cables; blue: polarized actin patches and cables; purple: abnormal actin cables and depolarized actin patches (N≥200 for each time point, 2 experiments - error bars show SD). (9.66 MB TIF) [file pone.0002556.s003.tif]

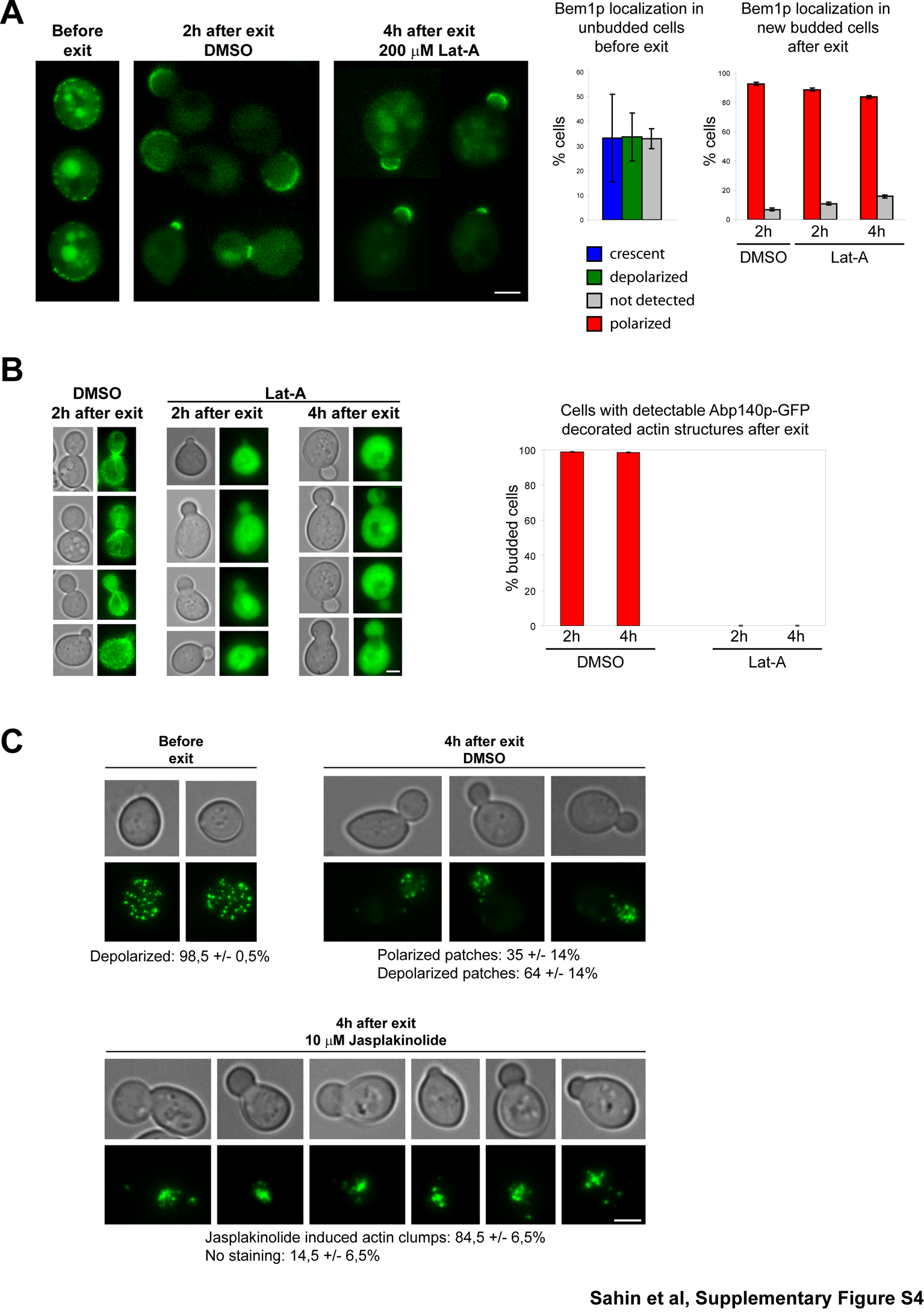

Supplement: Figure S4 — (A) Localization of Bem1p-3xGFP in wild type cells exiting from quiescence. Three copies of GFP were integrated at the BEM1 locus. This fusion protein is functional since it can be expressed in a bni1Δ or rsr1Δ without affecting their growth. Details of the construct are available upon request. Wild type cells expressing Bem1p-3xGFP were grown 7 days at 30°C, treated for 30 min with 200 µM Lat-A or DMSO. Cells were then re-fed with YPDA medium containing either 200 µM Lat-A or DMSO and grown at 30°C as described in material and methods. Bar 2 µm. Histograms display the percentage of cells with polarized Bem1p-3xGFP (red), depolarized Bem1p-3xGFP (green), Bem1p-3xGFP slightly polarized in a diffuse crescent shape manner (blue) or Bem1p-3xGFP not detected (grey). N≥200 for each time point, 2 experiments - error bars show SD. (B) Localization of Abp140p-GFP in wild type cells exiting quiescence in the presence of Lat-A. Wild type cells expressing Abp140p fused to GFP (Invitrogen, Carlsbad, CA) were grown 7 days at 30°C, treated for 30 min with 200 µM Lat-A or DMSO. Cells were then re-fed with YPDA medium containing either 200 µM Lat-A or DMSO and grown at 30°C as described in material and methods. Bar 2 µm. Histograms display the percentage of cells with detectable Abp140p-GFP decorated actin structures 2 or 4 h after exit from quiescence in the absence or in the presence of 200 µM Lat-A (N≥100 for each time point, 2 experiments - error bars show SD). (C) Cells exiting quiescence in the presence of jasplakinolide. Jasplakinolide sensitive strain (snq2Δ pdr5Δ erg6Δ see (Ayscough, 2000) expressing Abp1p-3xGFP from the endogenous locus (Sagot et al, 2006) were grown 7 days at 30°C. Cells were then pre-treated with 10 µM jasplakinolide or DMSO and then re-fed in YPDA medium containing 10 µM jasplakinolide or DMSO. Left panel: snq2Δ pdr5Δ erg6Δ ABP1-3xGFP after 7 days of growth at 30°C. Of note, because of the erg6 deletion, snq2Δ pdr5Δ erg6Δ ABP1-3xGFP cells do not display [file pone.0002556.s004.tif]
